# The compact beam energy measurement method of the photocathode RF gun by the solenoid and beam shaping

**DOI:** 10.1371/journal.pone.0314549

**Published:** 2024-12-03

**Authors:** Bin Sun, Binkang Li, Xinjian Tan, Xiufeng Weng, Yu Wang, Faquan Wang, Jianxin Zhang, Hongqiao Yin, Xiaodong Zhang

**Affiliations:** Northwest Institute of Nuclear Technology, Xian Shi, Shannxi, China; COMSATS University Islamabad, PAKISTAN

## Abstract

Beam energy, normalized emittance, and quantum efficiency are crucial parameters of the RF gun. In this study, we proposed a compact and low-cost method to measure the beam energy at the exit of the 120 MeV electron linac’s RF gun. We utilized a solenoid magnetic field to rotate an elliptical beam and measured the rotation angle of the beam to calculate the energy. To generate an elliptical electron beam, we inserted a slit device after the laser beam shaping aperture (BSA) to produce a long strip of driving laser beam for the RF gun photocathode. During the measurement process, we employed the Maximally Stable Extremal Regions (MSER) detection algorithm to measure the beam spot angle, improving the accuracy and stability of the angle measurement. This method does not require any changes to the accelerator lattice, nor does it require additional space. It only requires inserting a slit device to measure the beam’s energy. Our results indicated that the energy at the exit of the RF gun was 4-5 MeV, consistent with simulation calculations using ASTRA.

## 1 Introduction

**Fig 1:**
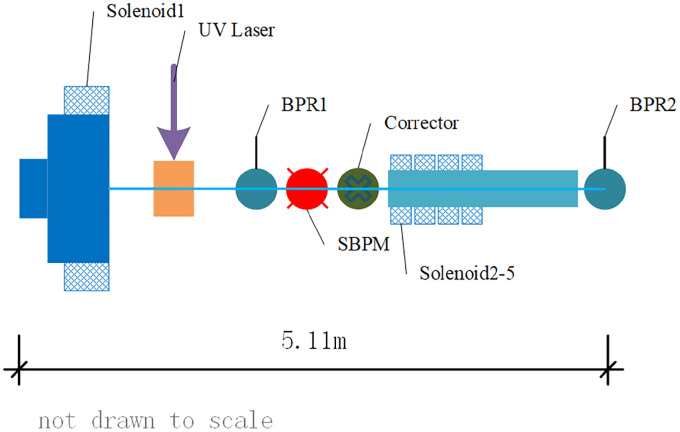
The measurement of RF gun layout.

High-quality electron beams are essential for a variety of advanced accelerator applications, including free-electron lasers, inverse Compton sources, and fourth-generation synchrotron radiation light sources. These applications demand beams with specific characteristics such as low emittance and energy spread to ensure high performance [1–3]. The 120 MeV electron linac, constructed in 2016, has been reliable for generating high-quality electron beams with the required low emittance and energy spread. As depicted in Fig 1, the lattice structure of this linac has been meticulously designed to meet these specifications. The operational parameters for the electron beam are defined with a charge range of 0.01 to 0.5 nC, a normalized emittance of less than or equal to 1.0 mm⋅mrad, and an energy spread of less than or equal to 0.3% (rms).

Over the seven years since the installation of the RF gun in 2016,the linac has demonstrated remarkable stability and success in high energy electron radiography [4], fast neutrons production via the electrodisintegration reactions of high energy electrons [5], measurement of undesirable neutron spectrum [6] and etc. With the ambition to enhance the capabilities further, there are planing to upgrade the energy of the linac from 120 MeV to 360 MeV. This upgrade requires a comprehensive reassessment of the linac’s key components, especially the RF gun, as detailed in Table 1.

**Table 1:** The parameters of the photocathode RF gun.

Parameters	Value
*Cathodematerial*	Cu
*Quantumefficiency*	> 3 × 10^−5^@266nm
*Peakworkingfieldintensityonthecathodesurface*	> 100MV/m
*beamenergyofRFgun*	4–5 MeV
*Beampulsewidth*(*FWHM*)	10 ps
*RFfrequency*	2856MHz
*Repetitionfrequency*	10 Hz
*RFpulselength*	3us

The beam energy measurement is a critical aspect of ensuring the linac’s performance meets the upgraded specifications. Traditionally, a dipole analysis magnet is used for such measurements, but this method can be constrained by the physical space available in the beamline. The need for high-precision measurements necessitates a deep understanding of the beam trajectory and the intricate magnetic field distribution of the dipole magnet, which can be challenging due to spatial limitations.

Solenoids are crucial components that serve multiple purposes, particularly in beam guidance, focusing, and stability.Solenoids have been increasingly employed for the optics control of low-energy beams [7]. Solenoids offer the dual advantage of focusing in both planes while maintaining the cylindrical symmetry of the system, which are used for emittance compensation. It is a technique to correct the emittance of the beam. This makes them ideal for measuring beam emittance and alignment of the beam trajectory, as demonstrated in various studies [8–13]. Building upon the pioneering work of Liao Shuqing from the China Academy of Engineering Physics, who tested the rotation beam method on a miniature linear induction accelerator [14, 15]. Pinayev from Brookhaven National Laboratory used a solenoid as a general-purpose measurement tool to measure electron beam energy, beam trajectory, and thermal emittance on the Coherent Electron Cooling (CeC) accelerator [16].

The proposed method refines the generation of elongated electron beams and the measurement of beam angles. It incorporates simulation experiments to provide a quantitative analysis of measurement errors. Furthermore, it employs the rotating beam method for measuring electron beam energy on the linac, all while scanning for the optimal RF phase for beam energy enhancement.

This approach represents an improvement over Liao Shuqing’s method which required modifications to the linac beamline to accommodate slit. Compared to Pinayev’s method, the proposed technique significantly reduces the number of samples needed from the beam image, from approximately 14–18 times to just 2–4 times. This reduction not only shortens the energy measurement time but also enhances the overall usability and efficiency of the algorithm, making it a valuable advancement in the field of electron beam measurement.

## 2 Beam energy measurement by solenoid

### 2.1 Measurement principle

According to beam transport theory, magnetic fields not only have a restraining effect on electron beams, but can also rotate them [12]. The angular velocity of electrons moving in a magnetic field varies with the change in the magnetic flux surrounding them [15].

$$\begin{array}{c} \varphi =\frac{e}{2m_{0}c\sqrt{\gamma^{2}-1}}\int_{l_{1}}^{l_{2}}B\left(z\right)dz \end{array}$$ (1)

*φ* is the rotation angle of the e-beam, *e* is the charge of the electron, *m*_0_ is the rest mass of the electron, *c* is the speed of light, *γ* is the Lorentz factor of the electron, and the integration is the solenoid magnetic field region that causes the beam to rotate. By calculating the rotation angle of the beam after passing through the solenoid magnetic field, the corresponding beam energy can be obtained from Eq 1 [17, 18]. From Eq 2, it can be seen that the error in energy measurement is mainly caused by three factors: the measurement error of the beam angle, the measurement error of the magnetic field strength, and other errors in the measurement system. The overall error can be calculated using Eq 2.

$$\begin{array}{c} \Delta_{\gamma }=\sqrt{\Delta_{B}^{2}+\Delta_{\theta }^{2}+\Delta_{s}^{2}} \end{array}$$ (2)

$\Delta_{B}=\frac{\Delta (\int_{l_{1}}^{l_{2}}B\left(z\right)dz)}{\int_{l_{1}}^{l_{2}}B\left(z\right)dz}$ is the integral error of solenoid,$\Delta_{\theta }=\frac{\Delta \theta }{\theta }$ is the error of the beam angle measurement, Δ_*s*_ is the system errors [19, 20].

### 2.2 Measurement method

The beam generated by the RF gun is generally axisymmetric and cannot be used to measure angular parameters. It is necessary to make the electron beam elongate with a certain directionality. To simplify the measurement system, a slit was installed after the laser BSA [21], cut the laser spot from a circular into a rectangular one, and generated an asymmetric electron beam that rotates at a certain angle through the solenoid. The image is displayed on the YAG screen downstream and recorded by a CCD camera. The rotation angle of the electron beam is measured by a stripe detection algorithm, and then the energy of the electron beam is calculated.

The method for measuring the beam angle is an important factor affecting the accuracy of energy measurement. The electron beam on the YAG screen is somewhat similar in distribution to the image of the image of the laser light stripe. The laser light stripe detection algorithm [22] can theoretically be directly applied to the measurement of the electron beam angle. However, in simulations and experiments, it was found that, due to the effect of space charge forces, it is difficult to generate a striped electron beam downstream of the linac, which is as narrow and elongated as the laser light stripe. An elliptical electron beam is produced at downstream, which requires higher demands on the angle detection algorithm. Conventional light stripe detection algorithms fail in this scenarios. Research has found that the detection algorithm for elliptical beam spot regions provides superior angle detection results. In this study, we selected the classic light stripe detection algorithm Steger [23] and the region detection algorithm MSER [24], and compared them with the conventional Gaussian fitting (CGF) method used in the Liao’s paper [15].

CGF is a classic algorithm for extracting the center of a light strip in images, which is based on the distribution of grayscale values and often assumes a Gaussian distribution. The algorithm performs a horizontal pixel density analysis at various vertical positions in the image to obtain the density distribution curves of the elliptical beam along the horizontal direction. After acquiring these curves, the coordinates of the highest area center point are determined by fitting them to a Gaussian distribution.

The Steger algorithm is another renowned method for light strip center extraction, capable of achieving sub-pixel positioning for structured light stripe centers, and is often considered the benchmark for such algorithms due to its high precision and stability. However, it involves computationally intensive convolution operations for each pixel, making it susceptible to environmental noise. It should be noted that the center points derived from both the CGF and Steger methods might not be linear, as shown in Fig 3e and 3f. To determine the direction of the electron beam, a linear fitting method is required. The RANSAC algorithm was employed to filter some outliers for more accurately calculating the direction of the electron beam.

The MSER algorithm is a well-established image processing technique for detecting text regions, primarily based on the watershed concept for spot area detection based on local image features. It draws on the idea of the watershed algorithm, identifying feature regions by finding extremal points in connected regions at different grayscale thresholds. These feature regions are considered stable within a certain range of grayscale changes and can serve as important features within the image. In the MSER algorithm, extremal regions are local maxima or minima in grayscale relative to their surrounding areas. These regions maintain their characteristics after the image undergoes affine transformations, including rotation, scaling, translation, etc. To achieve affine invariance, the MSER algorithm normalizes the size and corrects the rotation of the extracted extremal regions. Size normalization ensures consistency when comparing extremal regions of different sizes, while rotation correction ensures that the direction of the regions remains unchanged after transformation.

## 3 Simulation experiments in ASTRA

To verify the measurement accuracy of the beam angle detection algorithm for elliptical electron beam angle measurement, a simulation experiment was conducted in ASTRA, and the measurement accuracy was compared by calculating the energy.

**Fig 2:**
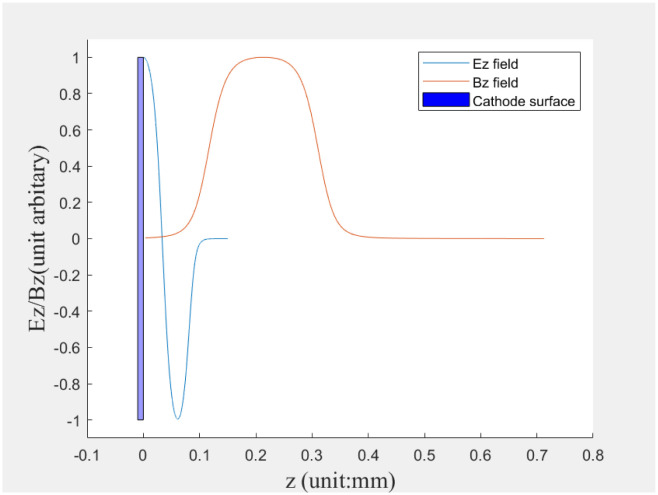
Overlap of the RF gun solenoid and accelerating cavity field.

The Astra setting parameters for the electron beam are initial sizes of x = 0.5 mm and y = 2.45 mm. Due to the slight overlap between the electron gun solenoid and the electron gun RF cavity, as shown in Fig 2, and the 120 MeV linac has solenoids installed on the accelerating tube, we set the tube RF off acting as a drift tube and used the solenoids on the accelerating tube as beam-rotating devices. The beam spot image is obtained at position BPR2, which be shown in Fig 1 at downstream z = 5.11 m. In the simulation experiment, a simulated CCD image is generated by sampling the results generated by Astra.

**Fig 3:**
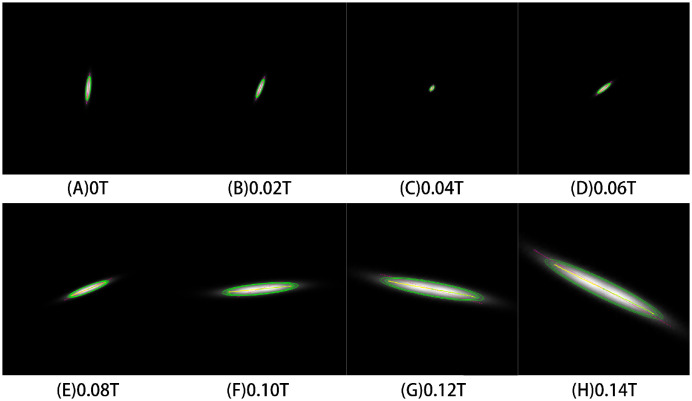
Simulation image and ellipse major axis detection results when different energy electron beams pass through the different solenoid strengths.

The energy estimation at the exit of the RF gun ranges between 4 and 5 MeV. The beam energies of the simulation experiments were set to 4.295, 4.792, 5.282 MeV. The solenoid magnetic field strengths were set to 0.00–0.2 T. The energy of the electron beam was calculated based on the angle measurement, and the results of the simulation experiment are shown in Fig 3. The red straight line is the beam angle measured by the CGF method, the yellow is the beam angle measured by the Steger algorithm, and the green is the beam angle measured by the MSER method, and the detailed results are shown in Table 2.

**Table 2:** Experimental results of simulation by ASTRA.

Beam Energy(MeV)	4.295	4.792	5.282
CGF(MeV)	3.700±0.715	3.465±1.745	6.474±3.299
Error(%)	13.841	27.692	22.567
Steger(MeV)	4.271±0.013	4.769±0.016	5.261±0.020
Error (%)	0.558	0.479	0.398
MSER(MeV)	4.271±0.019	4.772±0.016	5.260±0.020
Error (%)	0.558	0.417	0.416

By comparing the performance of three different angle detection algorithms, both the MSER and the Steger algorithm showed overall good results, while the CGF method exhibited a significant offset in the detection of beam angles from -45° to 45°. This is because the shape of the electron beam spot in this scenario is elliptical, which differs significantly from the typical application scenario of the CGF method, where long and narrow strips are typically used. Therefore, the CGF method had limitations and was not suitable for this scenario. Both the MSER and the Steger algorithm were very stable during the detection process and were able to accurately measure the beam angle. The average error in calculating the electron beam energy was less than 1%. As the beam changes shape under different solenoid strengths, the MSER algorithm, with its affine-invariant property, slightly outperforms the Steger algorithm. It should be noted that when the magnetic field strength causes the beam rotation angle to be greater than 180°, it becomes difficult to determine the accurate orientation of the elliptical major axis of the beam. In such cases, additional intermediate values may need to be determined to accurately determine the rotation angle.

During energy measurement experiments, the selection of an appropriate slit width necessitates a balanced consideration of cost, manufacturing complexity, and the precision of measurement. Common intuition suggests that a narrower slit could theoretically offer enhanced angular measurement accuracy, as the slit width is directly proportional to the energy’s precision. An overly narrow slit may diminish the electron flux, leading to reduced brightness on the YAG screen, which in turn lowers the signal-to-noise ratio of the captured measurement image and increases the manufacturing difficulty and costs. On the other hand, an excessively wide slit, while easing manufacturing demands and reducing costs, can result in a diminished aspect ratio that increases angular measurement errors.

**Fig 4:**
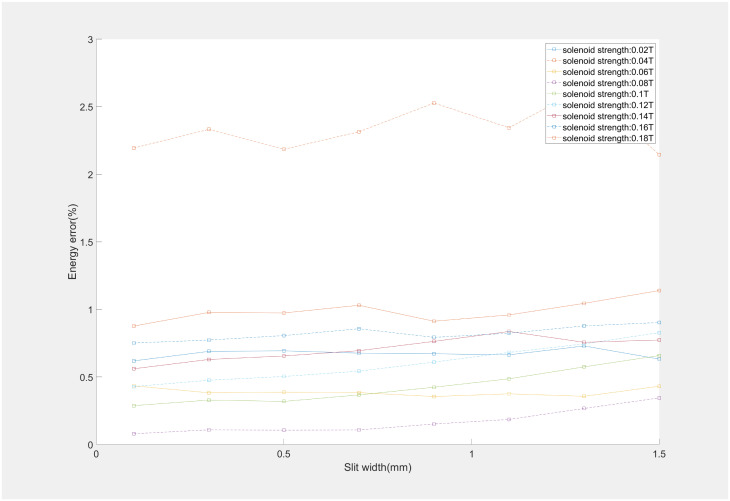
Correlation of different slit widths and the measurement energy error.

Utilizing the ASTRA can emulate the effects of varying slit width on beam energy measurements. The maximum height of a laser beam is determined by the diameter of a BSA. In these simulations, the initial height of the electron beam was set to 2.45mm alongside the different slit widths of 0.1 mm-1.5 mm to simulate the impact of different widths on the electron beams and to observe their influence on the energy measurement. The result was shown in Fig 4 at the 0.1 mm-1.5 mm slit widths.

The simulation results indicated that the MSER algorithm had stability and precision across energy measurements of beams with varying slit widths. The algorithm’s robustness is maintained even as the different slit widths. This stability is crucial for energy measurements, ensuring the reliability of data obtained under diverse experimental conditions. After simulation experiments, slit width of 0.5 mm was selected for the actual beam experiments, which is a balance between cost-effectiveness ie.difficulty in production and measurement precision.

**Fig 5:**
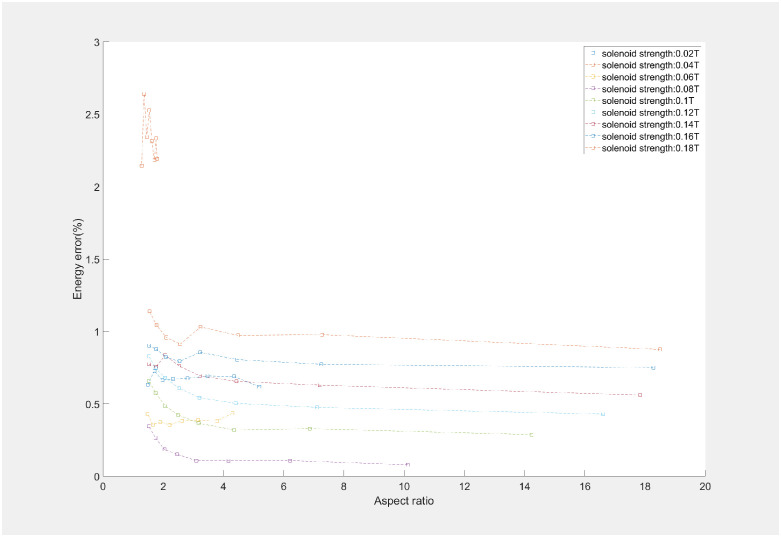
Correlation of different beam aspect ratios and the measurement energy error.

**Fig 6:**
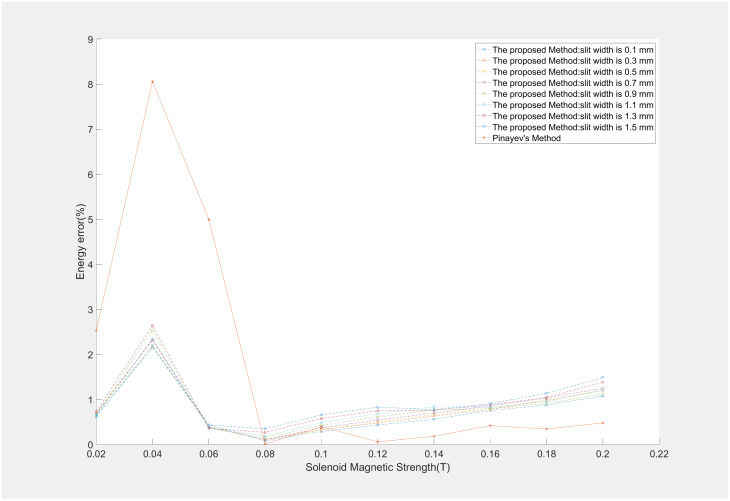
Comparation between the proposed methond with varying slit widths and Pinayev’s method.

An in-depth analysis was conducted in solenoid-based energy measurements through simulation experiments. The relationships between aspect ratio of the electron beam, solenoid strength, and energy measurement error were quantitatively delineated, as illustrated in Figs 5 and 6. The impact of solenoid strength on energy measurement precision significantly outweighs that of slit width and aspect ratio. Because variations in solenoid strength are directly linked to alterations in the beam rotation angle and shape. At a solenoid strength of 0.04T, the size of the electron beam was minimized, the aspect ratio was comparatively low, and the rotation angle was also small, leading to the highest energy measurement error. It was observed that an increase in slit width corresponds with a gradual increase in energy measurement error, a trend mirrored by changes in the beam’s aspect ratio. The predominant influencing factor is the solenoid’s strength, as the magnetic field intensity concurrently affects the aspect ratio and rotation angle of the beam. During the simulation experiment, the energy measurement error was found to be at its minimum when the solenoid magnetic field strength was at 0.08T, equating to an approximate rotation angle of 60°. We also presented a comparative analysis between the proposed method and Pinayev’s method. The method introduced in this work demonstrates equivalent precision to Pinayev’s approach at solenoid strengths below 0.1 T. With an increase in solenoid magnetic field strength, the beam shape becomes more dispersed, resulting in lower precision compared to Pinayev’s method. Nonetheless, the method proposed in this paper needed only two images per measurement, contrasting with the 12–18 images required by Pinayev’s method. The approach introduced here maintained commendable measurement accuracy while simplifying the measurement process.

In the simulation experiment, there was no error in the magnetic field region integration, and the energy error was caused by the angle measurement error. The average energy measurement error in the simulation experiment was approximately 0.5%. Therefore, it can be inferred that the angle deviation Δ_*θ*_ calculated by the MSER algorithm and the Steger algorithm is approximately 0.5%. According to data provided by the solenoid manufacturer, the measurement error of the magnetic probe is better than 1%, resulting in a Δ_*B*_ of approximately 1%. Therefore, by using Eq 2, the total measurement error can be controlled within approximately 1.5%.

## 4 Experimental results and discussion

**Fig 7:**
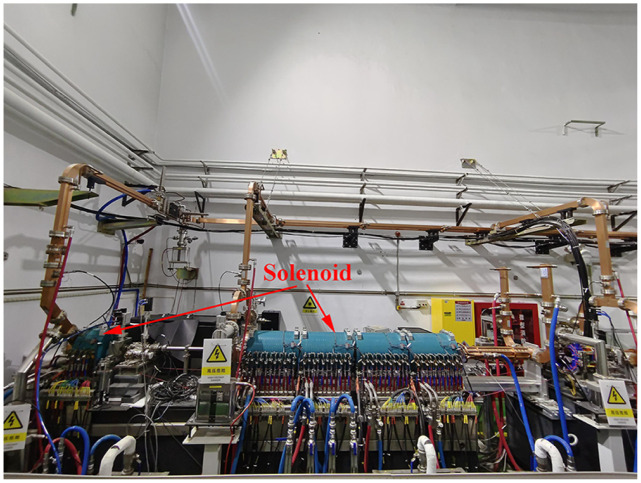
Solenoids on beamline.

In the 120 MeV electron linac, the dipole analysis magnet used for energy measurement is located at 15.26 meters downstream of the beam line, making it difficult to measure the beam energy at the exit of the RF gun. The solenoids are used to compensate for the increase in beam emittance caused by space charge forces, as shown in Fig 7. This motivated us to find a method of using the solenoid magnets to measure the beam energy after the RF gun. The blue part of Fig 7 is the solenoid around the accelerating tube.

**Fig 8:**
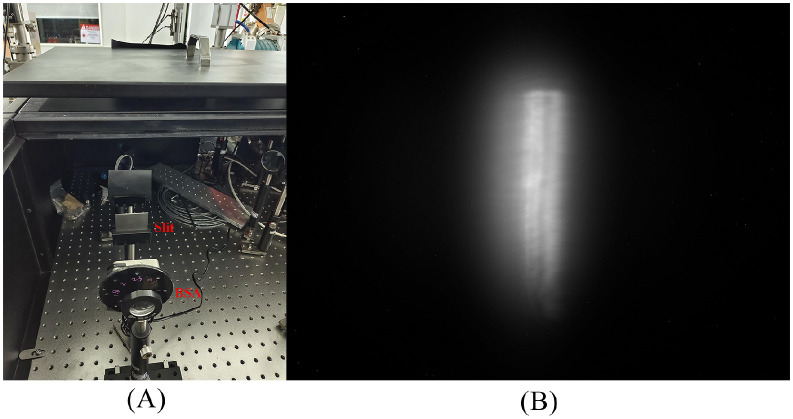
The slit device and the laser beam on the virtual photocathode.

In order to reduce the complexity of the measurement system, we proposed a method that inserting a 10 mm by 0.5 mm slit device after the laser BSA, as shown in left of Fig 8. After transmission, the laser beam spot size incident on the virtual photocathode was 2.45 mm by 0.5 mm, resulting in a long-strip laser beam as shown in the right of Fig 8.

The beam images at YAG crystals were captured by CCD cameras and analyzed to determine the angle of the beam. In addition, all the YAG crystals in the beam line were 10 mm by 10 mm. This area is in the range of the good field area of solenoid. The profile monitor has the advantage of allowing us to visually inspect images to ensure that they are well positioned within the YAG crystal area [16].

**Fig 9:**
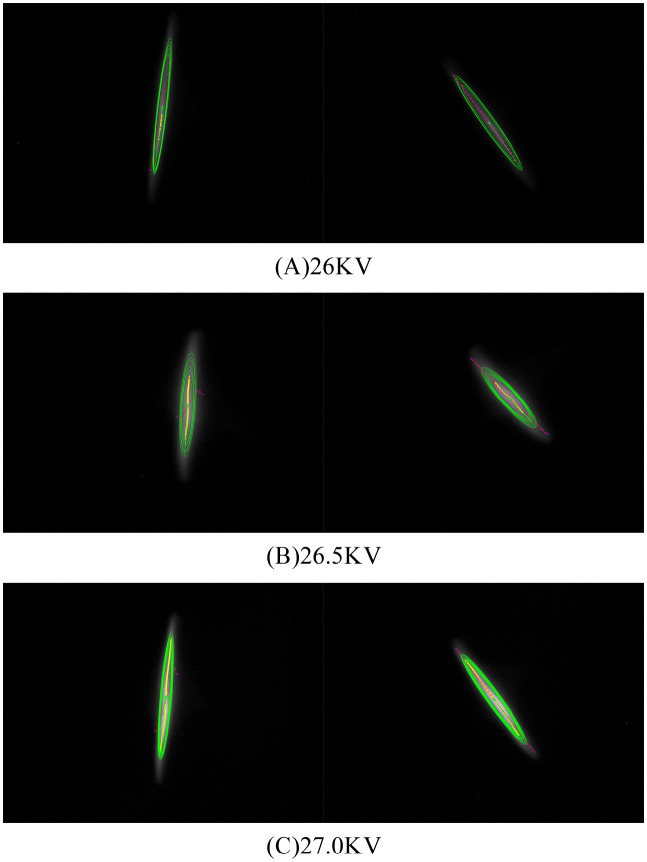
The angle detection results when different energy electron beams through the solenoid.

The magnetic field of the RF gun solenoid overlaps with the electric field of the RF gun accelerating cavity, and the power supply of the solenoid of the RF gun in the 120 MeV linac is not dual-polar. So we measured the beam energy using the second solenoid on the first accelerating tube which is RF off to act as a drift tube. The second solenoid uses a high-precision dual-polar power supply with ±30A, whose precision is better than 0.01%. The solenoid rotates the beam, with the direction of the rotation (clockwise or counterclockwise) dictated by the sign of the particle charge and the direction of the magnetic field of solenoid. The beam image captured by CCD cameras is shown in Fig 9.

**Fig 10:**
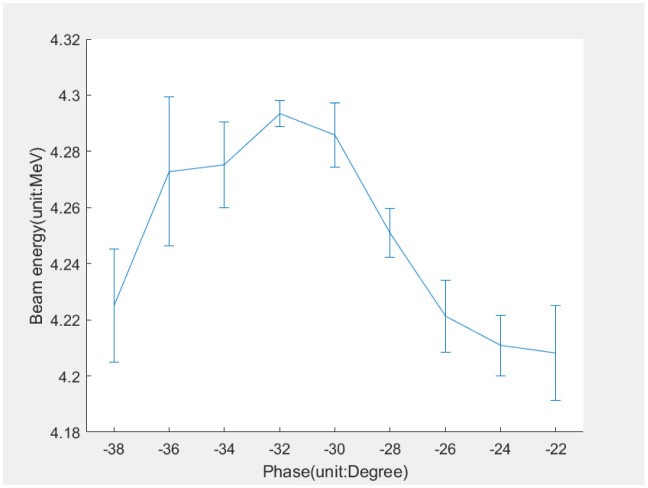
The correlation between phase and energy.

The measurement results of beam energy are shown in Table 3. At 26.5 kV of modulator, the Steger algorithm did not find a suitable beam axis and could not calculate the energy. The MSER algorithm was more stable and had better robustness. The CGF method had poor results, and both simulations and practical experiments proved its limitations in the elliptic beam spot. The experimental results showed that the electron beam energy at the exit of the photocathode RF gun was 4–5 MeV. We scanned the phase of the laser incident with the maximum beam energy at around -32 degrees from zero crossing, as shown in Fig 10.

**Table 3:** The results of energy measurement.

Modulator High Votage(KV)	Algorithm	Beam Energy(MeV)
26	CGF	2.237
26	Steger	3.756
26	MSER	3.775
26.5	CGF	3.691
26.5	Steger	——
26.5	MSER	4.141
27	CGF	3.198
27	Steger	4.263
27	MSER	4.267

The MSER algorithm is an image-based regional extraction technique grounded in local feature analysis, drawing inspiration from watershed segmentation methodologies. It pinpoints regions of interest by identifying extremal points within connected areas at varying grayscale thresholds. These regions are deemed stable across a spectrum of grayscale alterations, thereby constituting vital image features. The affine transformation equation is Eq 3.

$$\begin{array}{c} \left[\begin{array}{c} x_{1}\\ y_{1} \end{array}]=[\begin{array}{cc} a & b\\ c & d \end{array}]*[\begin{array}{c} x_{0}\\ y_{0} \end{array}]+[\begin{array}{c} e\\ f \end{array}\right] \end{array}$$ (3)

while *x*_0_,*y*_0_ are the original position, *x*_1_,*y*_1_ are the final position, *a*, *b*, *c*, *d* is rotations, scaling parameters, *e*, *f* is shift parameters.

In the study of beam transport, it is often necessary to process beam images to analyze and measure the characteristics of the beam. The beam transport process are calculated using the transfer matrix as Eq 4, they can be considered to have affine invariance.

$$\begin{array}{c} \left[\begin{array}{c} x\\ x^{\prime } \end{array}]=[\begin{array}{cc} R_{11} & R_{12}\\ R_{21} & R_{22} \end{array}]*[\begin{array}{c} x_{0}\\ x_{0}^{\prime } \end{array}\right] \end{array}$$ (4)

This means that no matter how the beams are transmitted through devices of the accelerator, their affine invariance features remain unchanged.

Within the MSER algorithm, extremal regions are characterized as local maxima or minima concerning grayscale values in relation to their immediate surroundings. After affine transformations, such as rotations, scaling, and translations, these regions preserve their inherent properties, thereby achieving affine invariance. To facilitate this, the MSER algorithm standardizes the size of the extracted regions and corrects their rotation. This size normalization ensures a uniform basis for comparison among regions of differing dimensions, while rotational correction maintains the regions’ orientation post-transformation. This indicates that the fundamental attributes of the beam imagery persist, irrespective of their passage through accelerator components. Such invariance is essential for accurate measurement and characterization of beam properties. The extremal regions isolated by the MSER algorithm can be advanced for feature matching and analytical purposes within the beam. For example, in beam transport experimental scenarios, the MSER algorithm can be instrumental in recognizing and monitoring shifts in the position and morphology of the beam.

## 5 Conclusion

In this paper, we proposed a compact method for electron beam energy measurement, utilizing the existing solenoid on the beamline, which is originally used for emittance compensation, to efficiently and accurately measure the electron beam energy without the need for additional dipole analysis magnets. Compared to Liao’s method, this approach places the slit in the laser beam path, eliminating the need for modifications to the beamline structure, thus reducing the implementation difficulty. Although the width of the beam spot increases, the use of the MSER algorithm as an angle detection method enhances the precision of angle detection. Compared to Pinayev’s method, the addition of a slit in the optical path greatly reduces the number of image samples required, with only 1/4 to 1/3 of the original method’s sampling times, thereby improving the stability of energy measurement. The ASTRA was used to simulate the slit width and beam aspect ratio, and comparisons were made with Pinayev’s and Liao’s methods. The ASTRA simulation experiments demonstrated that using the MSER algorithm to measure the long-axis angle of the elliptical beam spot is highly accurate, with an overall accuracy error of less than 1.5%. This paper also analyzes the similarities between beam transport matrices and affine transformations, deducing the reasons why the MSER algorithm, which has affine invariance, performs better in calculating different shape changes of the beam spot. In actual experiments, the measured electron beam energy is approximately 4–5 MeV, meeting the upgrade requirements. This energy measurement method is suitable for application in accelerators with spatial constraints. This approach has the potential to measure the energy of low-energy electron beams in various applications.

## Supporting information

S1 Data (ZIP)
