# Supplementary figures and images for: The compact beam energy measurement method of the photocathode RF gun by the solenoid and beam shaping
Source: PLoS One. 2024 Dec 3;19(12):e0314549. doi: 10.1371/journal.pone.0314549 (PMC11614267; doi:10.1371/journal.pone.0314549)

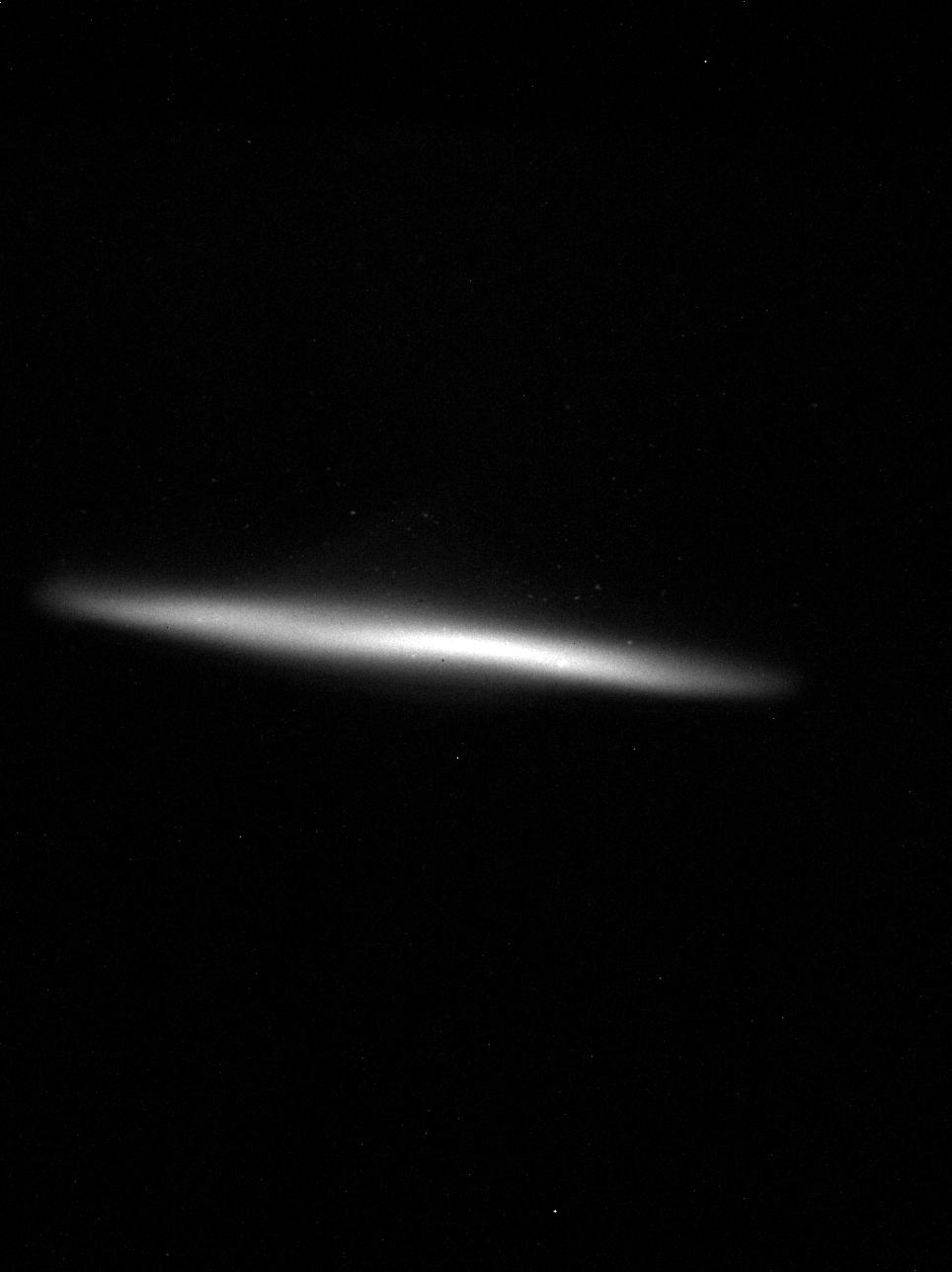

Supplement: S1 Data — (ZIP) [file pone.0314549.s001.zip › raw_data/26.5KV_401954.1333/26.5KV_401954.1333_-22.0103A.tiff]

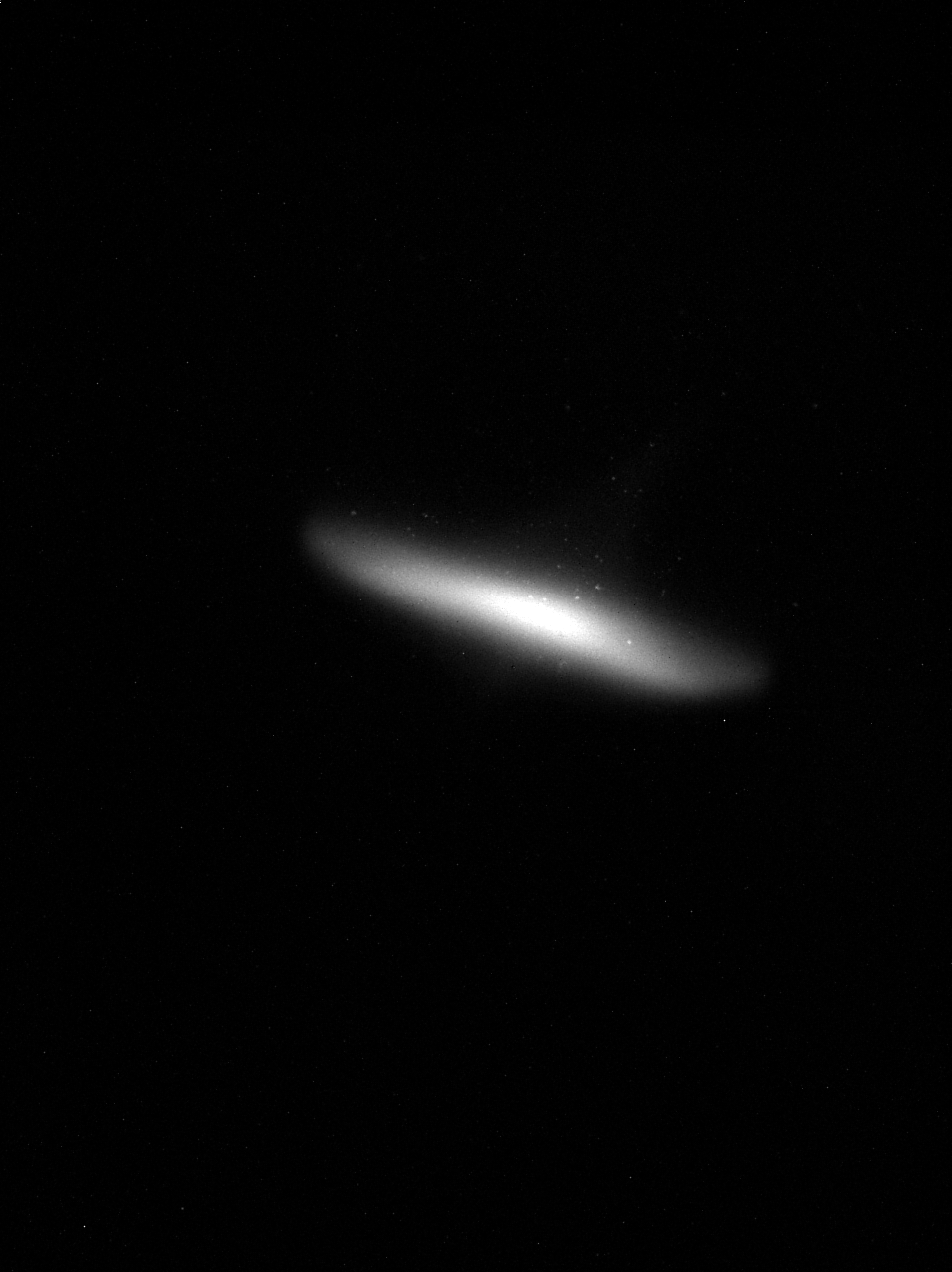

Supplement: S1 Data — (ZIP) [file pone.0314549.s001.zip › raw_data/26.5KV_401954.1333/26.5KV_401954.1333_-22.9896A.tiff]

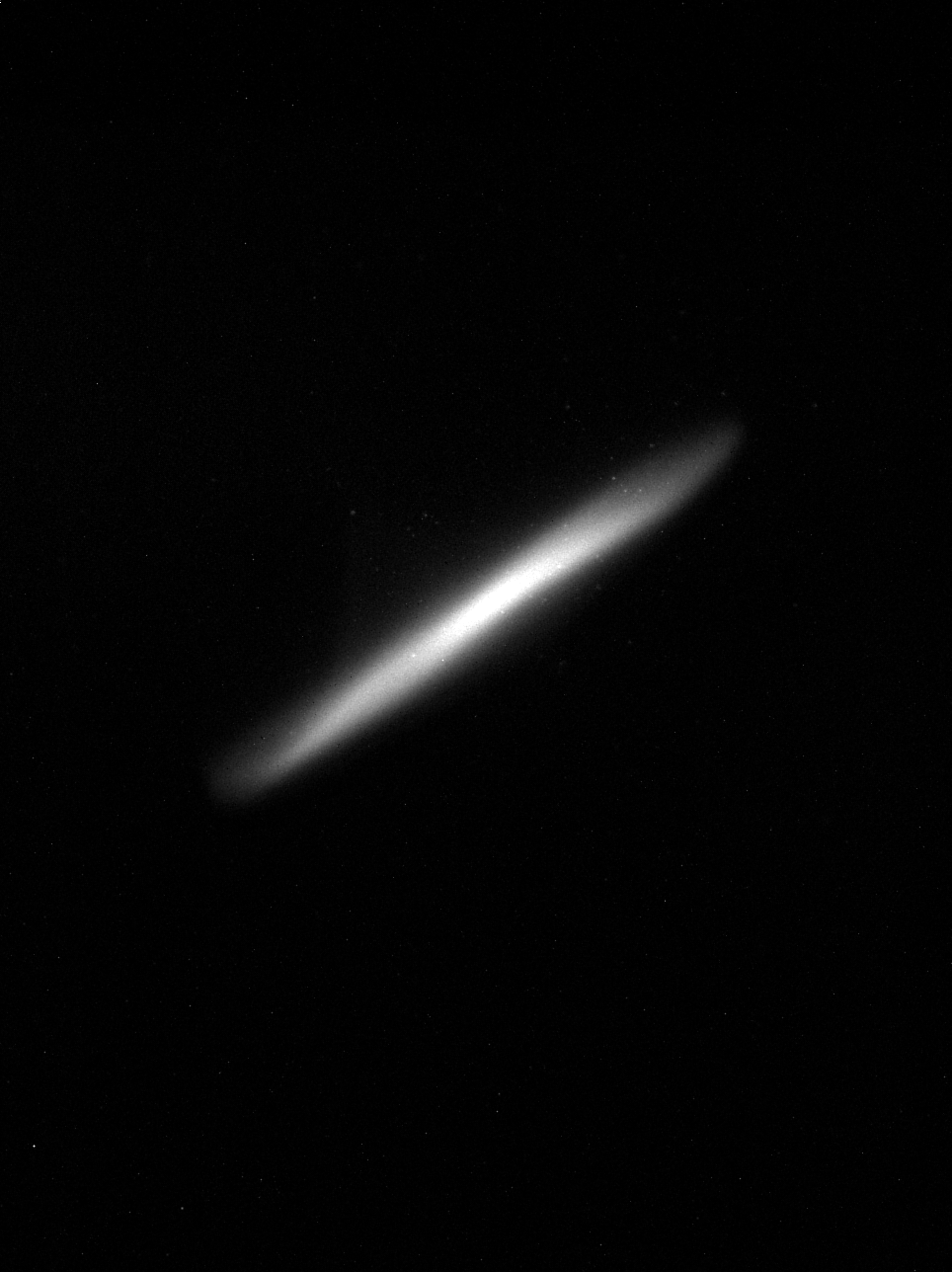

Supplement: S1 Data — (ZIP) [file pone.0314549.s001.zip › raw_data/26.5KV_401954.1333/26.5KV_401954.1333_21.9873A.tiff]

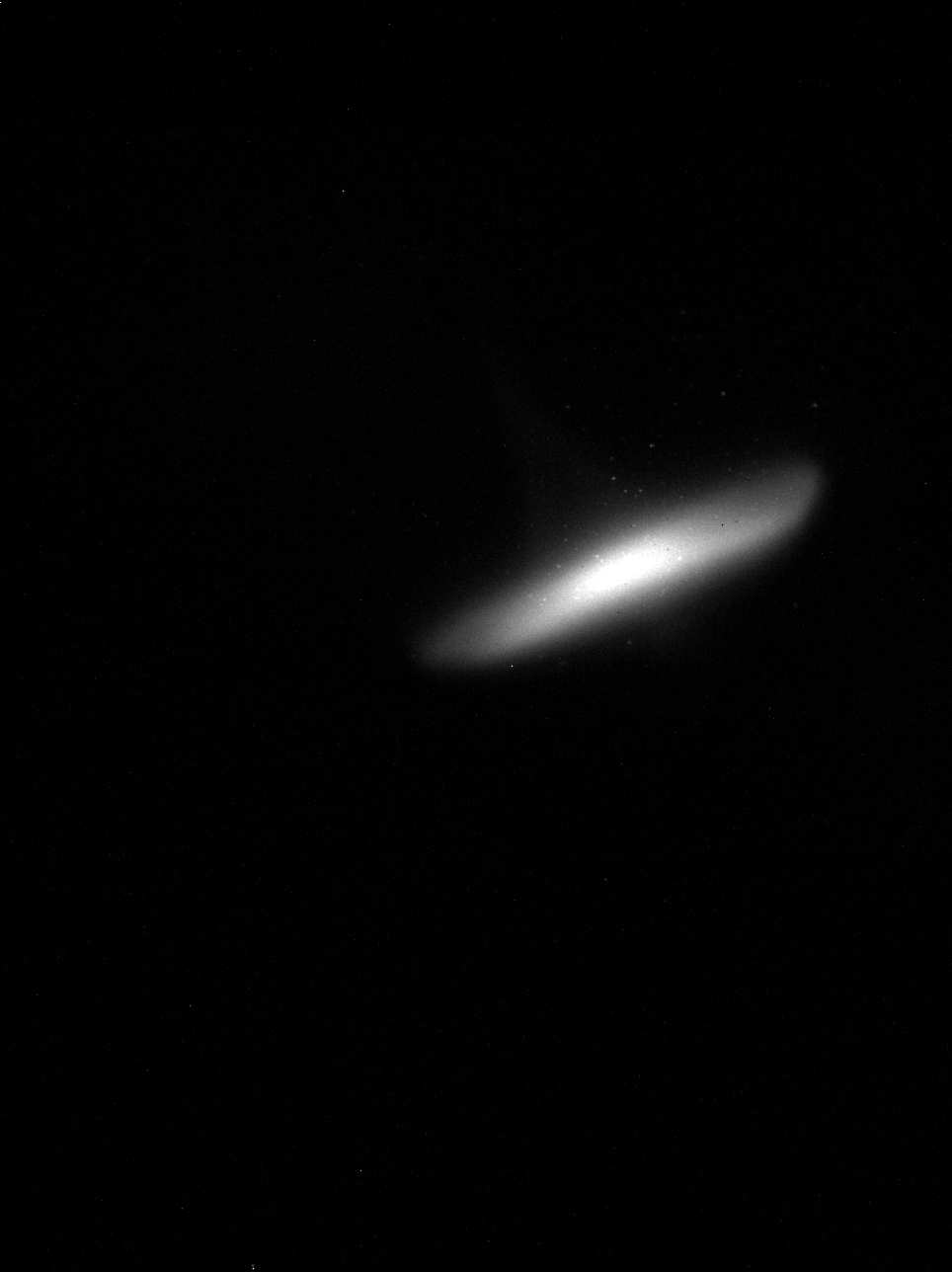

Supplement: S1 Data — (ZIP) [file pone.0314549.s001.zip › raw_data/26.5KV_401954.1333/26.5KV_401954.1333_22.9966A.tiff]

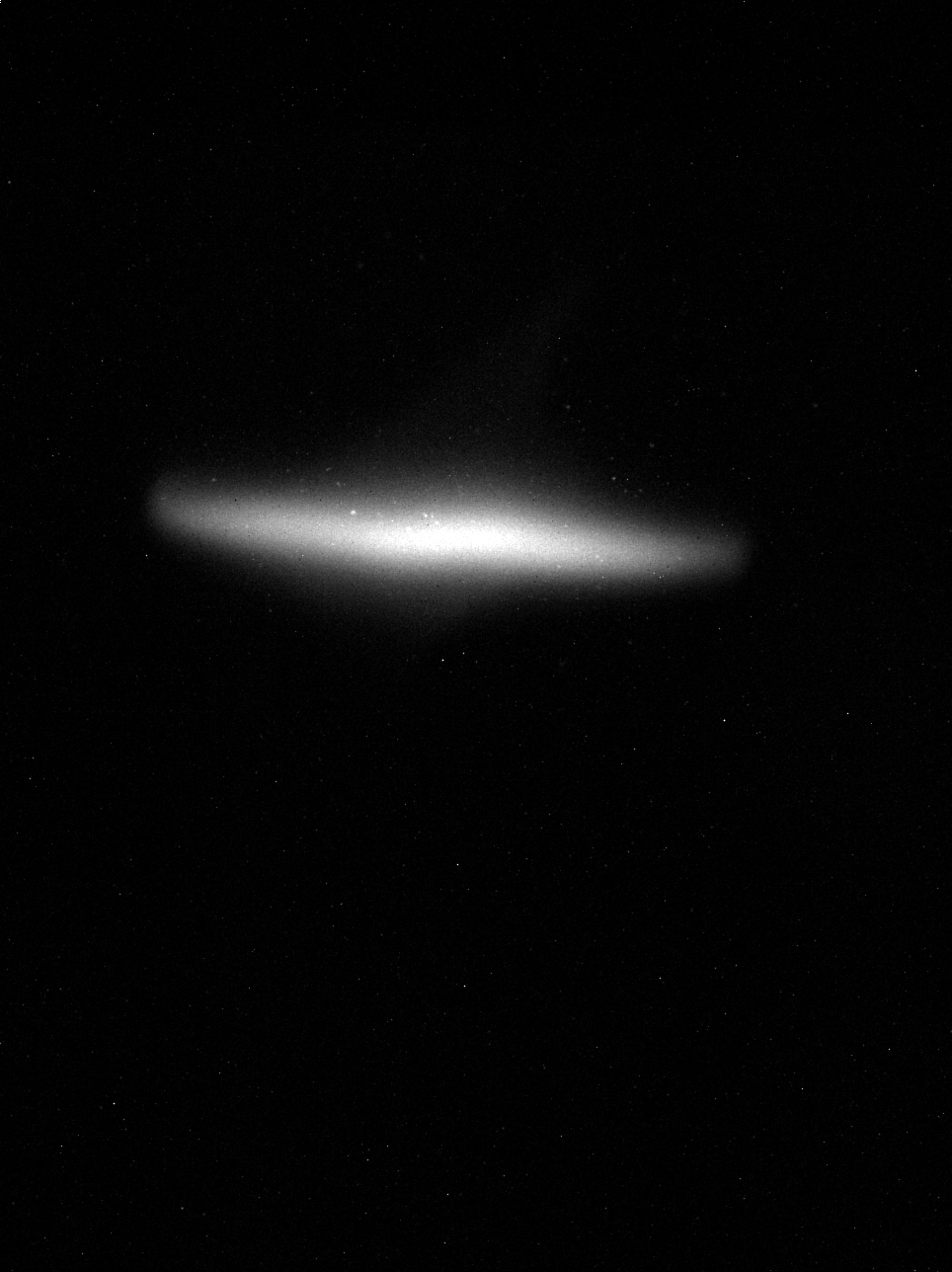

Supplement: S1 Data — (ZIP) [file pone.0314549.s001.zip › raw_data/26KV_401954.1333/26KV_401954.1333_-22.0054A.tiff]

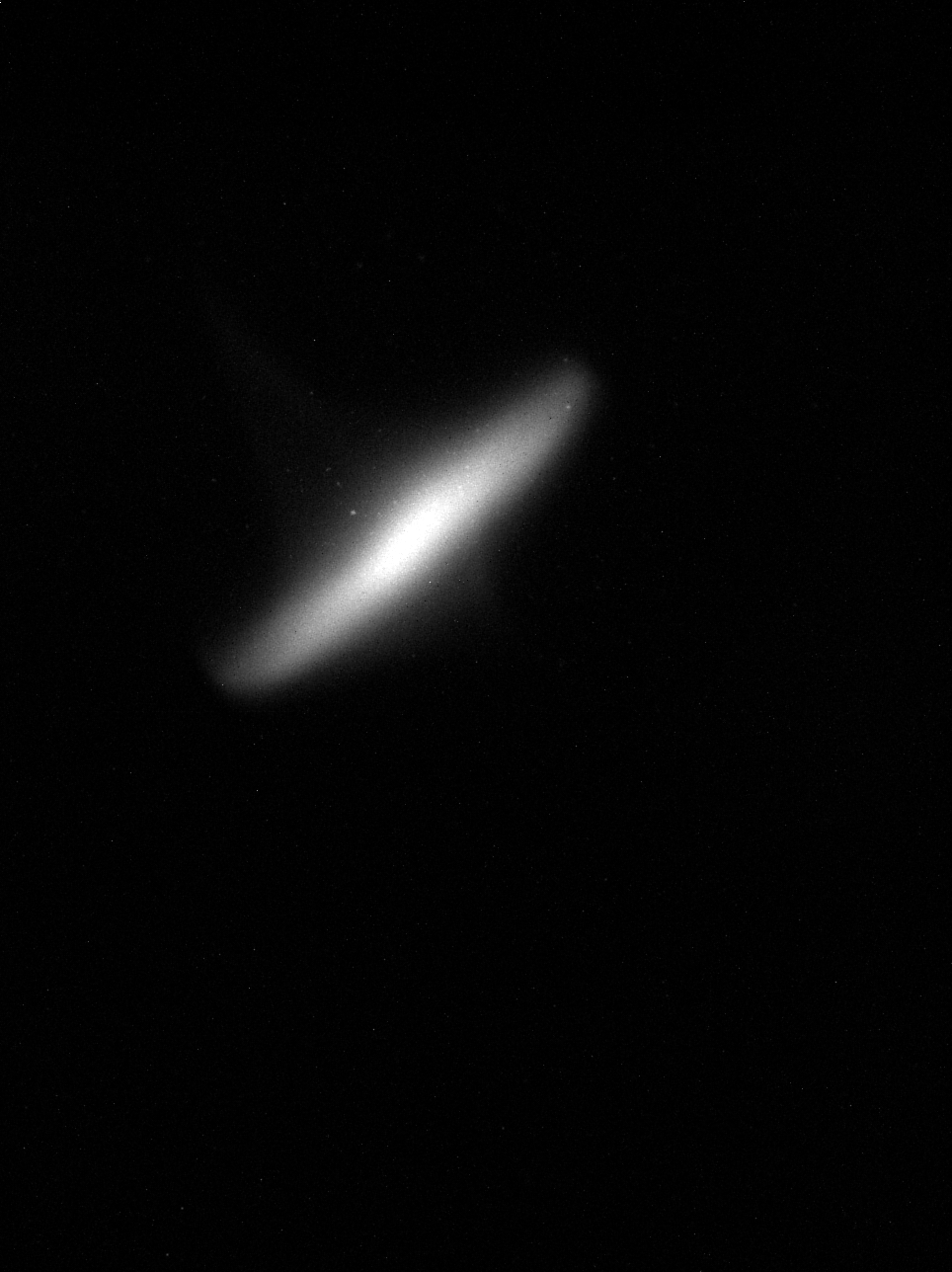

Supplement: S1 Data — (ZIP) [file pone.0314549.s001.zip › raw_data/26KV_401954.1333/26KV_401954.1333_21.9923A.tiff]

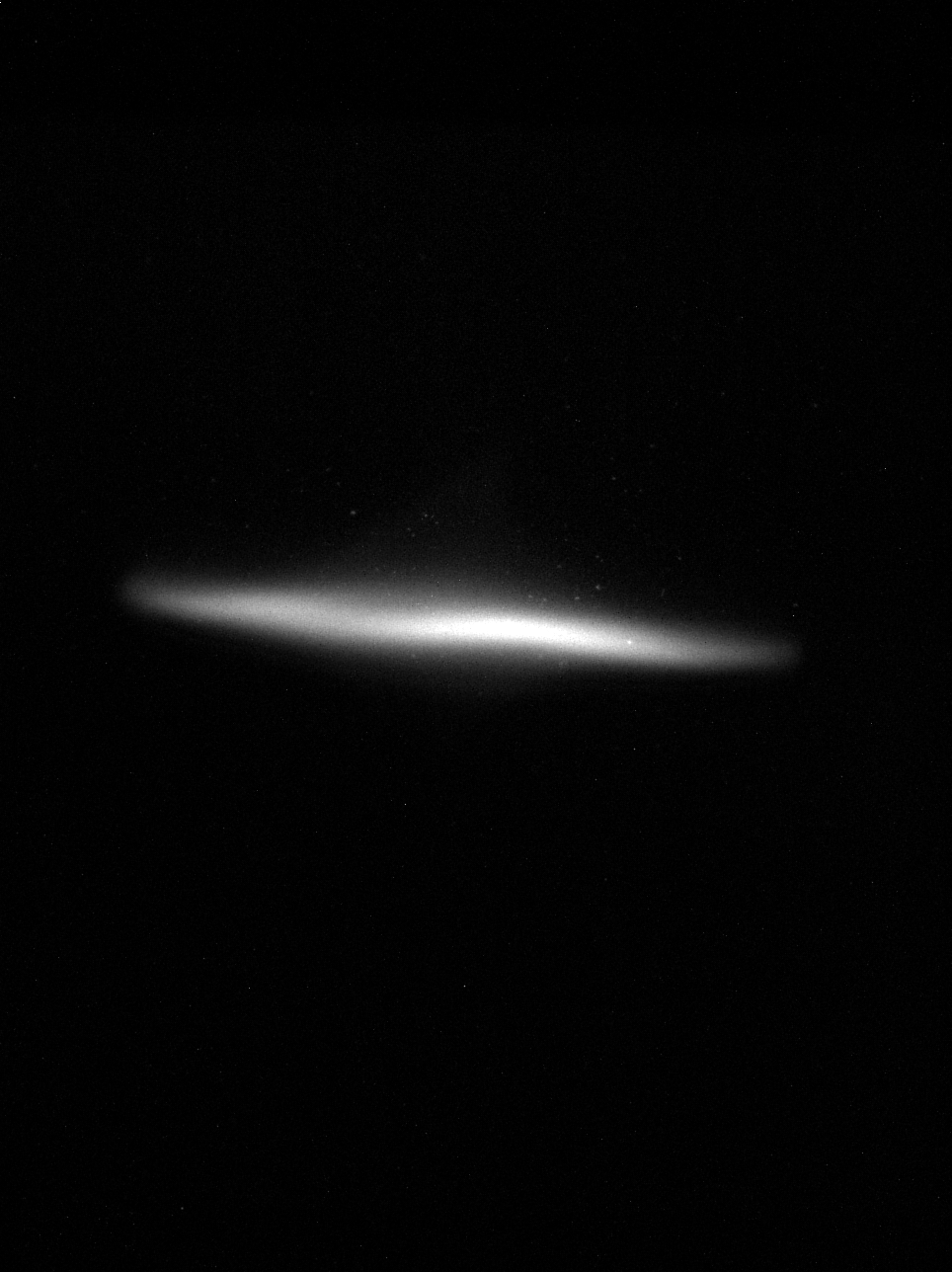

Supplement: S1 Data — (ZIP) [file pone.0314549.s001.zip › raw_data/27KV_401954.1333/27KV_401954.1333_-22.0096A.tiff]

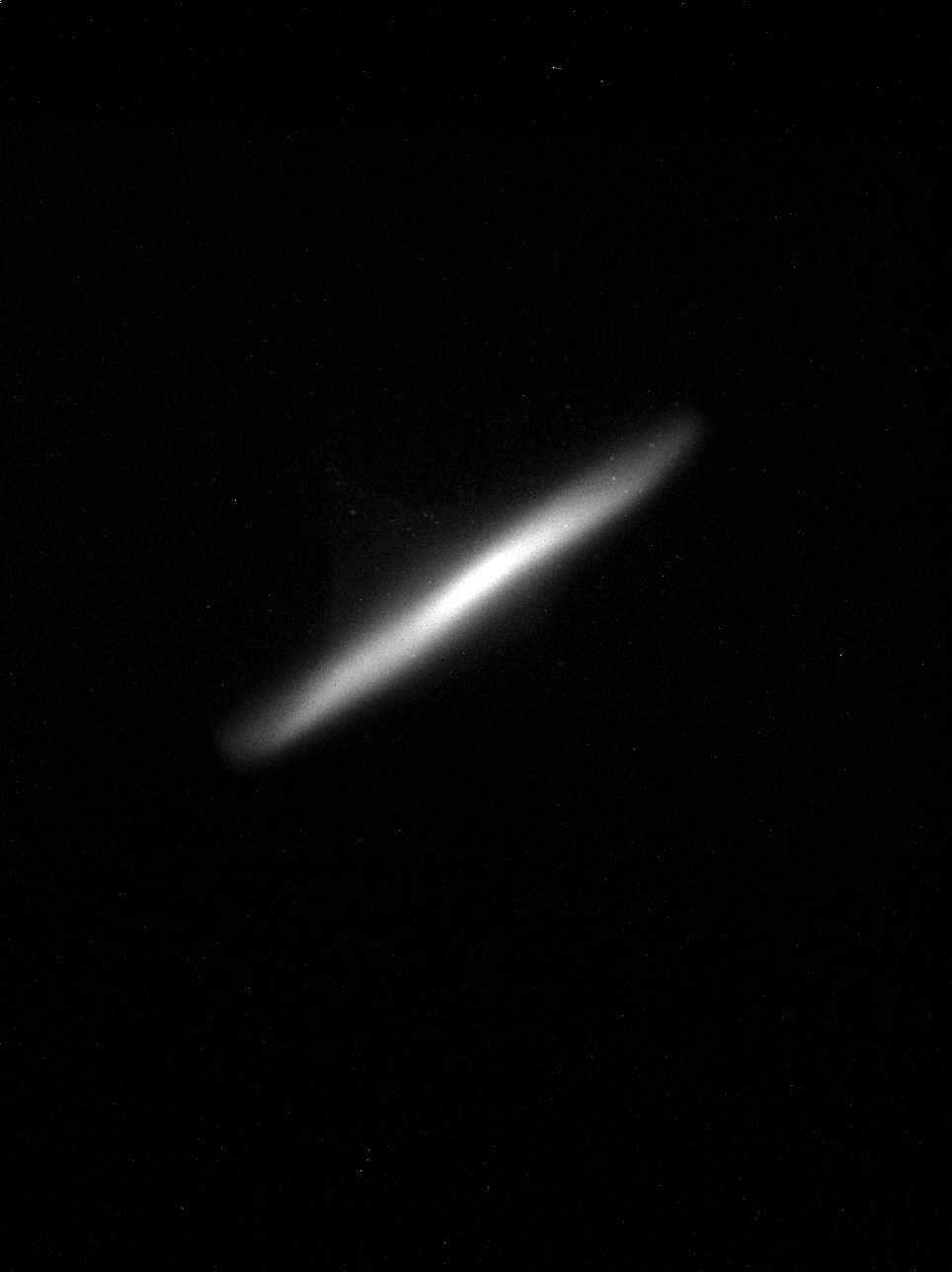

Supplement: S1 Data — (ZIP) [file pone.0314549.s001.zip › raw_data/27KV_401954.1333/27KV_401954.1333_21.9973A.tiff]
